# A focus on harnessing big data and artificial intelligence: revolutionizing drug discovery from traditional Chinese medicine sources

**DOI:** 10.1039/d3sc90185h

**Published:** 2023-09-26

**Authors:** Mingyu Li, Jian Zhang

**Affiliations:** a State Key Laboratory of Medical Genomics, National Research Center for Translational Medicine at Shanghai, Ruijin Hospital, Shanghai Jiao Tong University School of Medicine Shanghai China jian.zhang@sjtu.edu.cn; b Medicinal Chemistry and Bioinformatics Center, Shanghai Jiao Tong University School of Medicine Shanghai 200025 China

## Abstract

The advent of big data-driven artificial intelligence (AI) modeling has profoundly impacted the realm of drug discovery. Chen *et al.* (Q. Lv *et al.*, *Chem. Sci.*, 2023, https://doi.org/10.1039/D3SC02139D) have paved a way for modern drug discovery from traditional Chinese medicine (TCM) sources through their efforts over the past decade. They achieved this by creating TCMBank, the most extensive systematic central resource for TCM, which integrates standardized TCM-related big data and streamlines the AI-based drug discovery process.

## Abstract 2

Big data-driven AI modeling has greatly revolutionized drug discovery. Chen *et al.* have paved a way for drug discovery from traditional Chinese medicine (TCM) sources by developing TCMBank, a centralized resource which standardizes TCM-related big data and integrates an AI drug discovery pipeline.

## Graphical abstract

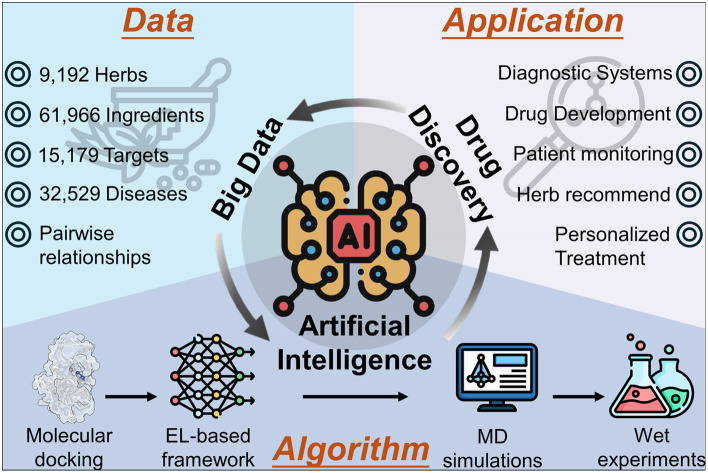

In the era of big data, data-driven AI modeling has revolutionized drug discovery, transitioning from serendipitous screening to rational design.^1^ The utilization of big data and AI in TCM stands as a quintessential example of this transformation, revitalizing the field and continuing to provide a reliable, abundant source for the development of modern pharmaceuticals.^2^

Historically, a significant proportion of medicines were derived from natural herbs. Upon gaining a comprehensive understanding of these herbs' effects, scientists dedicated substantial time and financial resources to high-throughput screening for the active ingredients responsible for their efficacy. Although progress has been slow in recent years, thousands of years of folk practice have explored a vast number of TCM. Over the past several decades, considerable efforts have focused on isolating active ingredients from TCM and investigating their potential targets, culminating in an enormous and intricate repository of TCM-related big data.^3^

The emergence of big data has opened up new possibilities for the modernization of TCM. However, there are several challenges to efficiently leveraging this data, collectively referred to as the “four Vs”: velocity, volume, variety and veracity. In terms of velocity, TCM-related information is expanding rapidly, and manual collection considerably lags behind the speed of data generation. Most existing TCM databases suffer from limited data volume, lack of data variety, and slow data velocity, with some not even being updated. This situation necessitates the use of advanced techniques to process data in near-real-time and effectively manage the continuous flow of information. Furthermore, traditional computational modeling methods for drug discovery may not be suitable for handling the vast amount (volume) and diverse types (variety) of data. In particular, when dealing with complex compound herbs and underlying biological mechanisms, the uncertainty (veracity) of the resulting data increases significantly.^4^ These challenges call for the development of innovative computational modeling methods to handle and analyze big data.

AI represents a feasible solution to these challenges, primarily due to its robust capacity to automatically capture underlying patterns within existing big data and use the patterns to predict new data.^5^ Data-driven modeling is essential for AI performance. This means that the size and quality of the training dataset heavily impact the accuracy of the models, with larger and higher quality datasets typically resulting in more accurate models. Moreover, a single model is more prone to overfitting. It may be too sensitive to specific information in the training set, leading to decreased prediction accuracy and difficulty in generalizing new, unseen data. To combat these issues, ensemble learning (EL) models are developed by combining multiple individual models to achieve better predictive performance and generalization ability.^6^

Therefore, at the core of TCM modernization is the collection of standardized TCM-related big data and the utilization of powerful artificial intelligence techniques that enable innovative modeling tailored to handle heterogeneous big data. Calvin Yu-Chian Chen and co-workers from Sun Yat-sen University have made remarkable strides in this field over the past decade. They have developed TCMBank, the most extensive systematic central resource for TCM (https://doi.org/10.1039/D3SC02139D).^7^ This database also incorporates an EL-based drug discovery workflow, which assists in identifying potential lead compounds and opportunities for drug repurposing.

Notably, to facilitate efficient big data collection and processing in TCMBank, an AI-based Intelligent Document Identification Module (IDIM) is developed. This module automatically gathers TCM-related information from various sources, including books, articles and TCM-related databases. After manual validation at least twice, a comprehensive TCM network was obtained, comprising 9192 herbs, 61 966 ingredients, 15 179 targets, 32 529 diseases, and their pairwise relationships.

The key submodule of IDIM is the biased LexRank module for automatic summarization of crucial sentences and keywords. The term “biased” refers to the incorporation of prior knowledge in the initialization weights to account for the original importance differences of critical sentences or words, instead of initializing all nodes with equal weight within classical LexRank.^8^ Prior knowledge for sentence summarization comes from feature fusion, where a multi-layer perceptron classifier is trained to predict the prior probability score of sentences being selected as summaries by using six predefined feature vectors. Subsequently, the entire document is converted into a graph, with nodes assigned the prior scores from the classifier. Edges are connected by the cosine similarity between nodes' feature vectors. Each node's score is updated iteratively, and summaries are generated using high-scoring sentences. Similarly, for keyword extraction, prior knowledge is incorporated with a word graph network derived from public dictionary data. Ultimately, the top *k* node words with higher node values are chosen as keywords. The feature fusion or prior graph-based biased LexRank has been validated for superior or comparable performance relative to other baselines on popular datasets and practical case studies. Importantly, by combining other AI techniques, such as selenium, pdfplumber, and optical character recognition, to regularly download and parse the latest PDF documents from PubChem, IDIM enables TCMBank to keep pace with the velocity of big data and continuous updates, significantly reducing labor costs.

After constructing TCM-related big data, Chen *et al.* further designed an EL-based drug discovery pipeline by combining molecular docking, EL models, molecular dynamics (MD) simulations, and experimental verification to accelerate drug discovery. The first step is to prepare the target protein sequence and structure, as well as ligand libraries from various sources. Next, Discovery Studio is used to compute and minimize the docking poses of ligands. After that, a ligand-based EL model is used for predicting the negative logarithm of their half-maximal inhibitory concentration (pIC50), which includes feature selection, 12 regression models, and a vote-average strategy. In parallel, a complex-based EL model is developed. This model encodes ligand–target pairs by integrating multiple deep neural networks to obtain embedding features, concatenating them, and finally decoding *via* fully connected layers to output affinity predictions. Resulting candidate ligands are assessed by combining docking scores, pIC50, and affinity predictions. They further utilize MD simulations and cell-based *in vitro* assays to verify the stability and functionality of ligand binding. The reliability of this EL-based drug discovery pipeline has been demonstrated by identification of potential inhibitors for colorectal cancer and Alzheimer's disease.^9,10^

Interestingly, although TCM is a part of natural products (NPs) and shares similar chemical or pharmaceutical properties, it still differs from NPs in certain aspects. Compounds in TCMBank exhibit a statistical trend of chemical properties that have longer tails (*e.g.*, an overdose of rotational bonds). Furthermore, a higher percentage of these compounds exhibit poor absorption, low solubility, and dose-dependent liver injuries, among other concerns. These observations suggest that TCM may not be intuitively friendly to the human body and should be used with caution.

Overall, the TCMBank by Chen *et al.*^7^ demonstrates how big data and AI can revolutionize drug discovery from TCM sources. The acquisition of adequate, highly reliable, and issue-specific big data (TCMBank) is a significant factor in the success of AI-assisted drug discovery. AI, in the form of IDIM, enables the constant updating of up-to-date big data, while the EL-based drug discovery workflow holds the potential to significantly enhance efficiency in promoting innovative and rational drug discovery, ultimately generating more high-quality data.

Integrating big data curation and advancements in AI research creates a sustainable paradigm widely applicable in drug discovery. Likewise, our lab has successfully developed the Allosteric database since 2009, using a combination of allosteric big data and AI-driven computational tools to transform the discovery of allosteric modulators from a serendipitous process to a more systematic and rational design.^11,12^ This has sparked significant pharmaceutical interest in the field. Moreover, as the large language models (*e.g.*, ChatGPT) continue to grow, incorporating them into the future of data-driven drug discovery holds potential for revolutionizing the field.^13^ However, the veracity of available data remains one of the formidable challenges.^1^ This is because data is heavily influenced by varying experimental conditions, especially when it comes to drugs operating within complex biological systems. In addition, more efforts are needed to increase AI modeling accuracy and robustness in diverse drug discovery settings. Meanwhile, resource sustainability issues are also becoming a concern in applying AI in drug discovery.^14,15^ Therefore, a revolution is needed not only in data utilization but also in methodological design.

## Author contributions

J. Z. conceived the project. M. L. and J. Z. wrote the manuscript.

## Conflicts of interest

There are no conflicts to declare.

## Supplementary Material
